# A human multi-cellular model shows how platelets drive production of diseased extracellular matrix and tissue invasion

**DOI:** 10.1016/j.isci.2021.102676

**Published:** 2021-05-29

**Authors:** Beatrice Malacrida, Sam Nichols, Eleni Maniati, Roanne Jones, Robin Delanie-Smith, Reza Roozitalab, Eleanor J. Tyler, Morgan Thomas, Gina Boot, Jonas Mackerodt, Michelle Lockley, Martin M. Knight, Frances R. Balkwill, Oliver M.T. Pearce

**Affiliations:** 1 Barts Cancer Institute, Queen Mary University of London, Charterhouse Square, London, EC1M 6BQ, UK; 2 School of Engineering and Materials Science, Queen Mary University of London, Mile End, London E1 4NS, UK

**Keywords:** biological sciences, cancer systems biology, cell biology, methodology in biological sciences, molecular biology

## Abstract

Guided by a multi-level “deconstruction” of omental metastases, we developed a tetra (four cell)-culture model of primary human mesothelial cells, fibroblasts, adipocytes, and high-grade serous ovarian cancer (HGSOC) cell lines. This multi-cellular model replicated key elements of human metastases and allowed malignant cell invasion into the artificial omental structure. Prompted by findings in patient biopsies, we used the model to investigate the role of platelets in malignant cell invasion and extracellular matrix, ECM, production. RNA (sequencing and quantitative polymerase-chain reaction), protein (proteomics and immunohistochemistry) and image analysis revealed that platelets stimulated malignant cell invasion and production of ECM molecules associated with poor prognosis. Moreover, we found that platelet activation of mesothelial cells was critical in stimulating malignant cell invasion. Whilst platelets likely activate both malignant cells and mesothelial cells, the tetra-culture model allowed us to dissect the role of both cell types and model the early stages of HGSOC metastases.

## Highlights

• Platelets are associated with a poor prognosis tissue composition
• A 3D tetra-culture tissue model enables dissection of platelet action in metastasis
• Platelets stimulate mesothelial and tumor cells to produce a diseased matrisome
• Platelet activation of the mesothelium permits tumor invasion

## Graphical abstract

**Figure:**
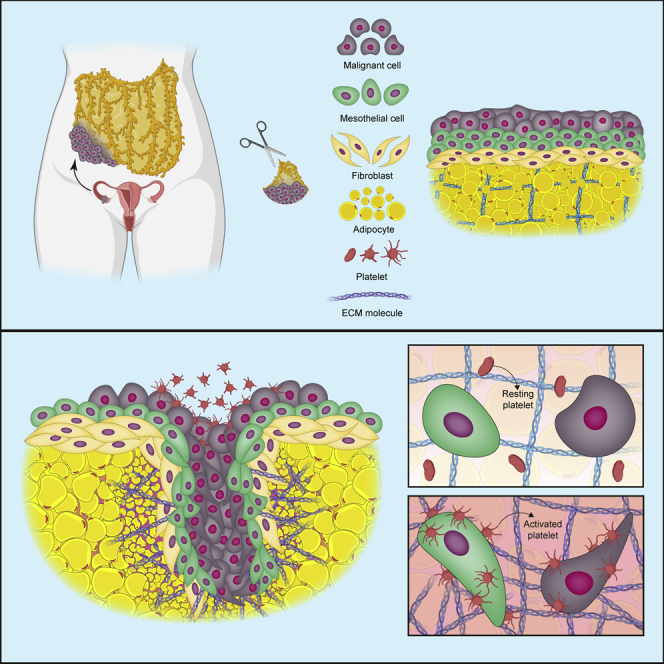

## Introduction

Various components of the tumor microenvironment (TME) can act as barriers to effective cancer treatments through suppressive or protective cell phenotypes, the upregulation or activation of resistance pathways, or through the composition of the acellular microenvironment. In the latter, the tumor extracellular matrix (ECM) has been identified as a major barrier to effective cancer treatment, including checkpoint immunotherapies (Mariathasan et al., 2018; Tauriello et al., 2018), cell therapies (Anderson et al., 2017), and small molecule chemotherapies (Kalluri, 2016). Whilst every invasive tumor will have a disease-associated ECM, the composition of the ECM is both prognostic (Pearce et al., 2018) and predictive (Chakravarthy et al., 2018) of therapy response, suggesting some ECM compositions are more tumor promoting than others. The ECM is also important in the establishment of the metastatic niche through the interaction between circulating tumor cells and the microenvironment of a distant site, which can determine the success of colonization and establishment of a metastatic lesion. Combination therapies that target global deposition of ECM, such as TGFβ inhibitors (Mariathasan et al., 2018; Tauriello et al., 2018), or specific ECM components, for example *in situ* degradation of hyaluronic acid, can improve therapy response in murine cancer models (Hingorani et al., 2018). However, the tumor ECM can also be tumor-inhibitory by containing the malignancy and slowing disease progression (Cox and Erler, 2016). Therefore, deconvoluting ECM production from tumor inhibiting vs tumor promoting may identify approaches to inhibit tumor metastasis and improve tumor response to emerging and established cancer therapies.

The aim of our research is to use human multi-cellular models to study cancer-associated ECM. Our approach was to first ‘deconstruct’ the human cancer tissue that we wished to study, the commonly occurring omental metastases of high-grade serous ovarian cancer (HGSOC) (Pearce et al., 2018). This multi-level analysis at the biomechanical, cellular and molecular level provided us with a template and validation for ‘reconstruction’ of the tissue *in vitro*. Using this template we developed a tetra-culture model comprising human primary omental adipocytes, fibroblasts, mesothelial cells and early passage HGSOC

cell lines. We have used this model to investigate the role of platelets in the early stages of metastasis, tissue invasion and the production of a tumor supportive ECM.

High platelet levels in blood are correlated with a significant poor prognosis in several cancer types, including ovarian cancer (Stone et al., 2012). We wished to see if our multi-cellular human *in vitro* model could add further mechanistic information to previous *in vivo* findings on the role of platelets in driving disease progression, metastasis and tissue invasion that have been studied in murine models (Cho et al., 2017; Haemmerle et al., 2016, 2017; Hu et al., 2017; Labelle et al., 2011, 2014).

We found that platelets stimulate malignant cell invasion, ECM remodeling and production of ECM molecules associated with poor prognosis in the tetra-cultures. We then investigated the cell types involved in these actions of platelets. Previous experiments in mouse cancer models found that platelet activation of malignant cells leads to the generation of an EMT phenotype which drives tissue invasion during the early stages of metastasis (Labelle et al., 2011). The human tetra-culture model allowed us to dissect the role of individual cell types in a way that would not be possible in mouse cancer models. We found that platelet activation stimulated an EMT phenotype in malignant cells, but this was sufficient to stimulate their invasion into the artificial omental contruct. Instead we found that platelet activation of mesothelial cells was essential for malignant cell invasion within our model. Whilst platelets likely activate both malignant cells and mesothelial cells in disease, the tetra-culture model presented here has allowed us to dissect the role of both cell types and we propose that it is activation of the mesothelial cells that is most important in driving early stages of omental metastasis of HGSOC.

## Results

### Design and establishment of a multi-cellular model of HGSOC metastasis

**Figure 1:**
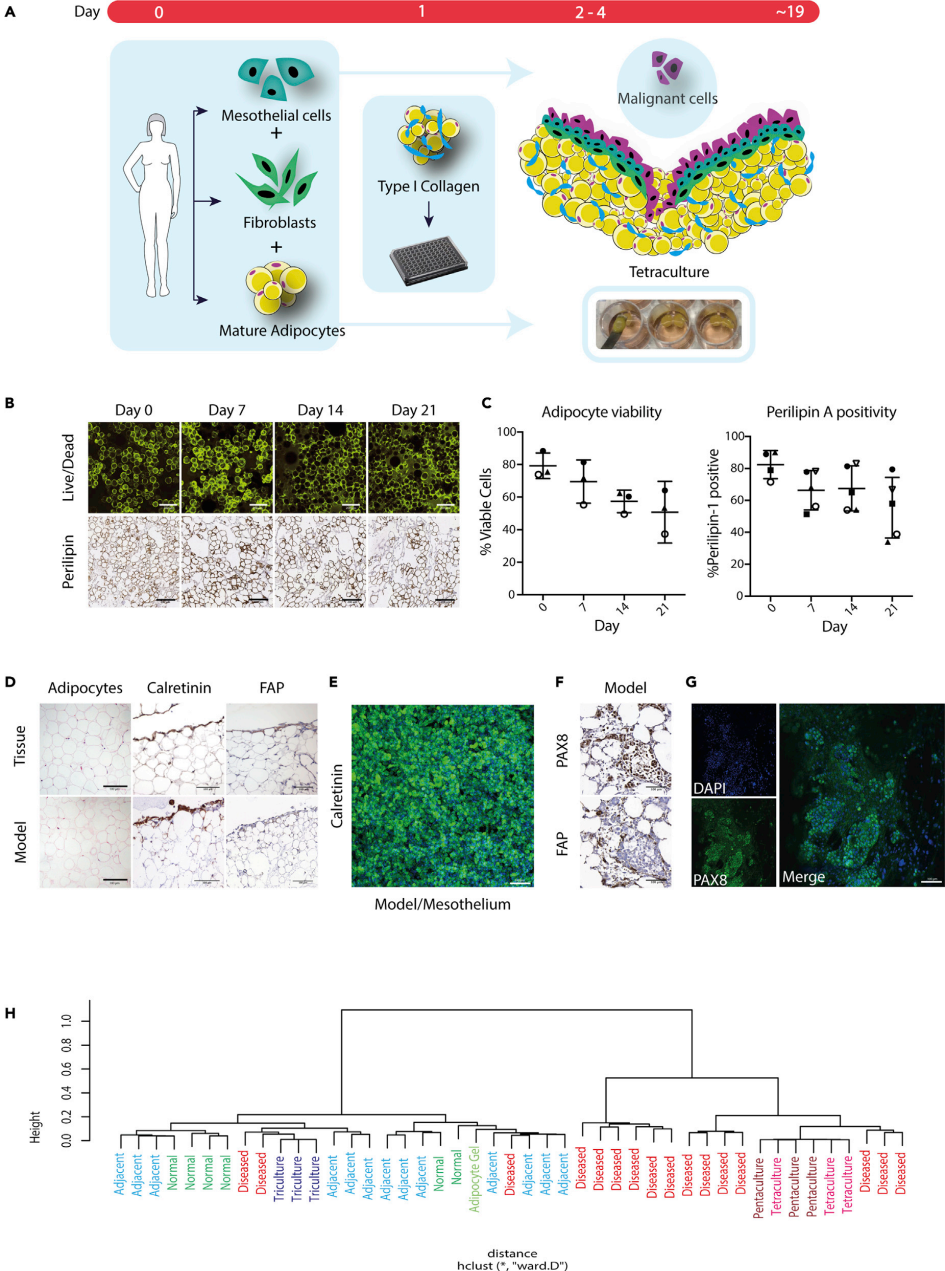
Tri- and tetra-cultures are viable and resemble the structure of human omental metastasis (A) Schematic figure shows the tri- and tetra-culture setup. (B and C) (B) Tri-culture viability was recorded for 21 days using live/dead staining assay and confocal imaging (upper panels) and perilipin metabolic adipocyte staining for immunohistochemistry (IHC) (lower panels) (scale bar 200 μm). The IF and IHC positivity for at least 3-5 gels for each time point was then quantified and plotted. Data are represented as mean ± SD (C). (D) H&E and IHC staining using calretinin for the mesothelial cells and FAP for fibroblasts reveal that the structures of the tri-culture and the human tissue are comparable (scale bar 100 μm). (E) IF staining of the surface of the tri-culture model shows a uniform deposition of mesothelial cells (scale bar 100 μm). (F) Representative image of the surface of tetra-culture gels stained for PAX8 and FAP that show that AOCS1 and fibroblasts form colonies on the surface of the tetra-culture which are mimicking human omental metastatic structures as shown in Figure S1 (scale bar 100 μm). (G) IF staining for PAX8 reveals that G164 cells adhere to the surface of the tetra-culture forming aggregates similar to those seen in human metastatic tissues (scale bar 100 μm). (H) Hierarchical cluster analysis of tetra- and penta-culture illustrates that both cluster more closely with historic HGSOC RNASeq (Pearce et al., 2018) tissue than normal or adjacent omental tissues.

In our previous work we deconstructed a metastatic site of high-grade serous ovarian cancer (HGSOC) (Pearce et al., 2018), finding that omental tissue with low or no disease present was primarily constructed of mature adipocytes with a surface layer of fibroblasts and mesothelial cells. Using a proteomic approach, we also determined that the major component of the ECM (Naba et al., 2017) was collagen 1, which we found made up over 50% of the tissue, and in some cases over 90% of the tissue. Therefore, to establish a low or no disease human omental tissue model, freshly isolated primary omental adipocytes were set within a 3D substrate of high-density collagen 1 to make an adipocyte-collagen gel monoculture. Next, primary omental fibroblasts were added to the surface of the adipocyte-collagen gel, followed by primary omental mesothelial cells to generate a multicellular tri-culture that replicates some elements of the normal omentum. An overview of the process is shown in Figure 1A. We then assessed the viability of the adipocyte cultures over a period of 21 days using a fluorescent live/dead cell stain and determined that there was a 20%–30% reduction in adipocyte viability over 21 days (Figures 1B and 1C left panel). This was also confirmed using perilipin A as a marker of adipocyte metabolic activity (Figure 1C right panel). From these data, we established that the tri-culture models were viable and suitable for up to 21 days of co-culture. Visual comparison with human omental tissue free from malignant disease (Figure 1D) showed that the tri-culture recapitulated the normal tissue morphology in the arrangement of adipocytes (Figure 1D left panels), the localization of calretinin staining for mesothelium (Figure 1D middle panels and Figure 1E), and the localization of FAP + fibroblasts (Figure 1D right panels).

Next we asked if we could use this cell model to study malignant cell invasion. We added HGSOC cell lines AOCS1 (Milagre et al., 2015) (Figure 1F) or G164 (Tamura et al., 2020) (Figure 1G) as a single cell suspension, creating an omental metastasis tetra-culture model. We found that both AOCS1 and G164 malignant cells attached to the surface of the cultures forming colonies (Figure 1G), similar to what we observed in patient tissues of early metastatic lesions (Figure S1A), where they could be clearly defined from other cells present in the model using nuclear PAX8 as a marker.

Finally, we extracted RNA from the cultures and used RNAseq to investigate the transcriptomic profiles of tri- and tetra-cultures and their similarities to human omental tissue with high or low/no metastasis. We found that tri-culture of adipocytes, fibroblasts and mesothelial cells clustered with human omental tissue samples with no or low disease whilst the tetra-culture with HGSOC malignant cells clustered with heavily diseased human omentum samples (Figure 1H). Taken together, these data show that a viable and relevant multi-cellular model of early omental metastasis model can be prepared in semi-high throughput manner that partially self-arranges at the cellular level. Next, we tested findings from our previous HGSOC ‘deconstruction’ study using the model.

### Platelets and TGFβ signaling are associated with the tumor ECM and poor prognosis

**Figure 2:**
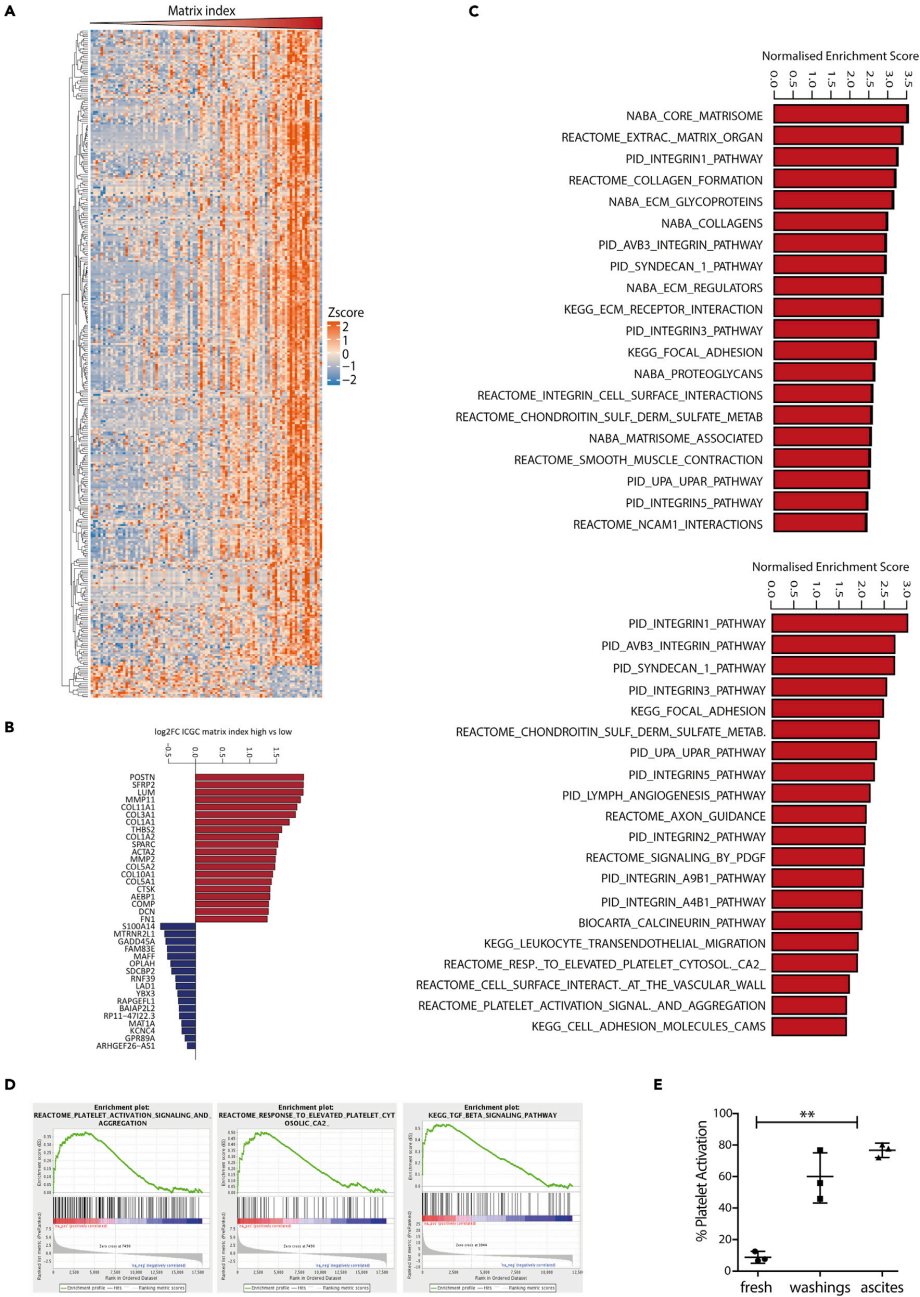
Platelets signaling is associated with MI and poor prognosis (A) Heatmap of differentially expressed genes in matrix index high vs matrix index low samples (adjusted p value < 0.05, NMIhigh = 21, NMIlow = 72). Samples (columns) have been ordered by increasing matrix index value. Genes (rows) have been clustered based on a pearson's correlation matrix as distance and the complete clustering method. (B) Differential expression analysis was performed on the high versus low matrix index samples. Barplot illustrates top up and down-regulated genes (adj. p value < 0.05). (C) GSEA normalized enrichment scores (NES) for significantly upregulated signaling pathways between high and low matrix index in human tissue (FDR <0.05). (D) Enrichment plots from the GSEA analysis showing a strong correlation between the MI signature and platelets and TGF-beta signaling pathways. (E) Flow cytometry analysis of CD62P⁺ activated platelets in fresh blood, washing with physiologic solution and ascites fluid from patient diagnosed with stage 3 and 4 HGSOC (n = 3) (∗∗p < 0.01 one way ANOVA). Data are represented as mean ± SD.

In our previous work we identified an ECM expression signature, MI, that associates with poor prognosis and immunosuppressive cell phenotypes across 13 different human cancer types, including HGSOC (Pearce et al., 2018). Using the ICGC HGSOC transcriptional database, we investigated the differentially expressed genes between patient samples that had high vs low MI scores (Table S1, Figures 2A and 2B). We observed major changes in ECM pathways (Figure 2C top panel) such as those involved in collagen formation or ECM regulation. Using KEGG pathway analysis for biological processes, we also found strong associations between the MI and platelet activation (Figure 2C bottom panel and 2D) confirming data published that associated platelets with tumor progression in ovarian cancer (Stone et al., 2012). In concordance with this, we analyzed the platelet content of fresh blood and ascites and confirmed that platelets are present predominantly in an activated state within the ascites fluid from patients diagnosed with Stage 3/4 HGSOC (Figure 2E). Since platelets have been correlated with a number of different processes including extravasation, establishment of the metastatic niche, stimulation of an EMT phenotype and anoikis inhibition (Cho et al., 2017; Haemmerle et al., 2016, 2017; Hu et al., 2017; Labelle et al., 2011, 2014; Laubli and Borsig, 2010), we decided to use the tetra-culture model to test whether platelets could stimulate ECM deposition and malignant cell invasion.

### Platelet addition associates with ECM remodeling to a poor prognosis tumor matrisome

**Figure 3:**
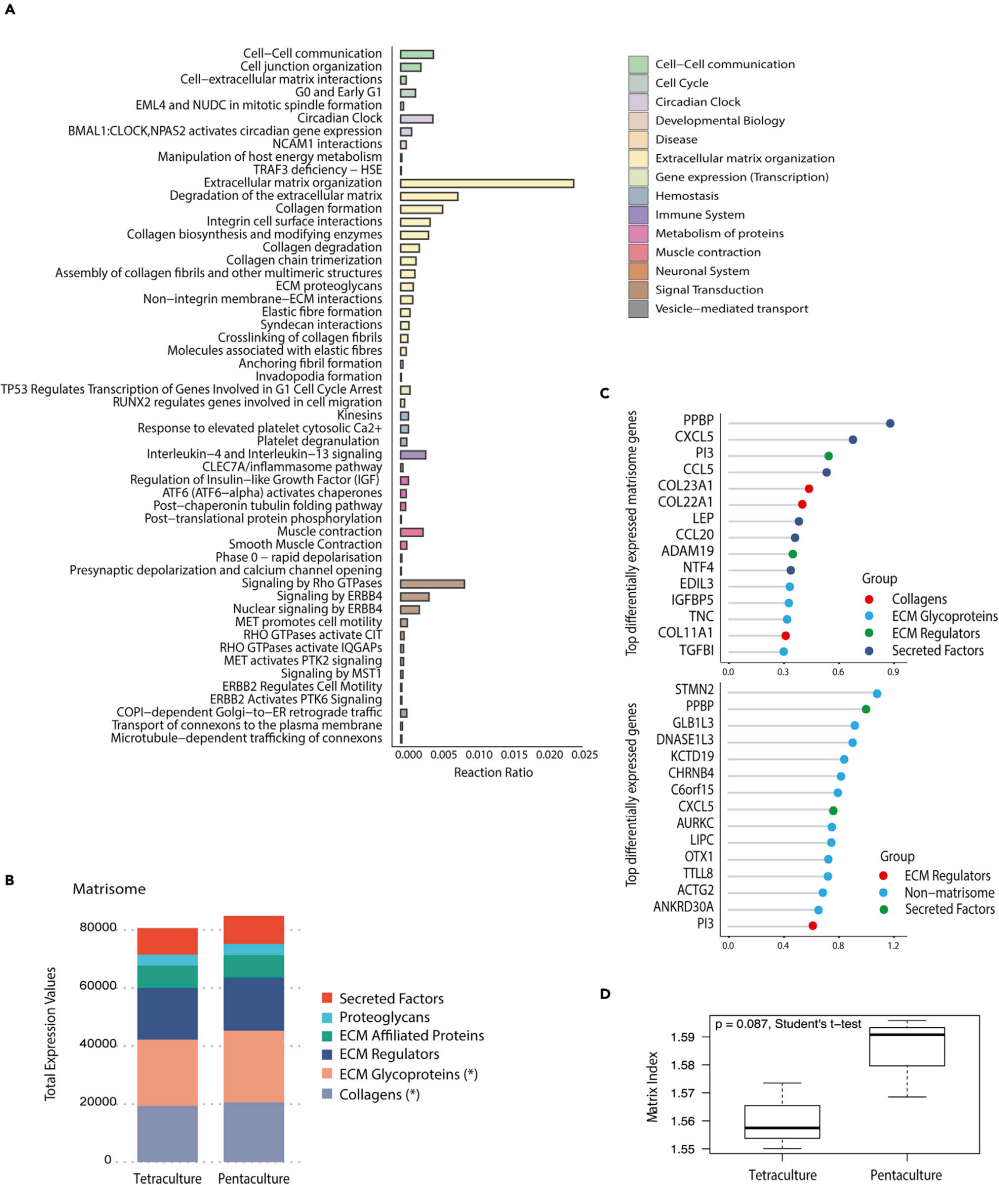
Platelet addition to the tetra-culture model stimulates ECM production and alters ECM composition RNAseq was performed on tetra- and penta-cultures (n = 3 in each group). (A) Significantly enriched Reactome pathways (p < 0.05) in penta-cultures compared to tetra-cultures include ECM organization, immune system and hemostasis. (B) Analysis of matrisome compartment identified a significant increase in expression of glycoproteins and collagens, ∗ = p < 0.05. (C) Top differentially expressed matrisome genes and all genes between penta- and tetra-cultures include *COL11a1*, *TNC*, and *TGFBI* (p < 0.05). (D) Boxplot showing median and interquartile range of MI in tetra- and penta-cultures (n = 3 in each group).

To test whether platelets were stimulating production of the disease-associated matrisome found previously (Pearce et al., 2018) and in Figure 2, we added human platelets to the tetra-culture to make a “penta”-culture. After 10 days of culture, we compared the transcriptional changes in the tetra- and the penta-culture using bulk RNAseq and compared the results to patient HGSOC omental metastases. The transcriptomic profiles (Tables S2 and S3, and Figures S2A–S2D) showed that the tetra- and penta-cultures clustered together and also clustered more closely with diseased HGSOC tissue than more normal or adjacent-to-tumor omental tissues (See penta-culture in Figure 1H). We then compared tetra-culture and penta-culture transcriptomic profiles using reactome pathway analysis (Table S4 and Figure S2E), which showed ECM organization pathways as the most highly enriched when platelets are present (Figure 3A shows the pathways involved in ECM organization; 15 of a possible 18 pathways significant). Other significantly enriched pathways in the penta-cultures containing platelets were immune system (cytokine production, most likely triggered through platelet action), post-translational modifications, hemostasis (indicative of the presence of platelets including platelet degranulation, and response to elevated platelet cytosolic Ca^2+^), smooth muscle contraction (that is associated with stromal cell activation in response to tumor invasion), and circadian clock (Figure 3A).

To further explore how platelets may be altering ECM organization, we used the transcriptomic data from tetra- and penta-cultures to look at the composition of the matrisome between these two conditions. We observed a significant expansion in the glycoprotein and collagen compartments of the matrisome in the penta-cultures (Figure 3B), which we had previously observed in our comparison between low and high diseased human HGSOC tissue (Pearce et al., 2018). We also assessed the top genes differently expressed between penta- and tetra-cultures (Figure 3C), where we identified several ECM molecules previously associated with poor prognosis in cancer (*COL11A1*, *TNC*, *TGFβ1*). Other top differentiated genes that resulted from the presence of platelets included cancer-associated inflammatory cytokines *CXCL5* and *CCL5* which are associated with invasiveness, metastasis (Romero-Moreno et al., 2019; Singh et al., 2018) and platelet guided formation of a metastatic niche (Labelle et al., 2014). Since the addition of platelets within the model stimulated predominately an ECM remodeling signature, we wondered whether platelets were also altering expression of the genes identified in our previously reported prognostic MI signature (Pearce et al., 2018). Comparing tetra-with penta-cultures, we saw an enrichment of the MI in penta-cultures (Figure 3D). Overall, these transcriptional data indicate that platelets are stimulating the production of a tumor ECM which has previously been associated with poor prognosis in HGSOC, with the formation of a metastatic niche, and with malignant cell invasion within tissues.

### Platelets stimulate ECM protein production within the 3D model

**Figure 4:**
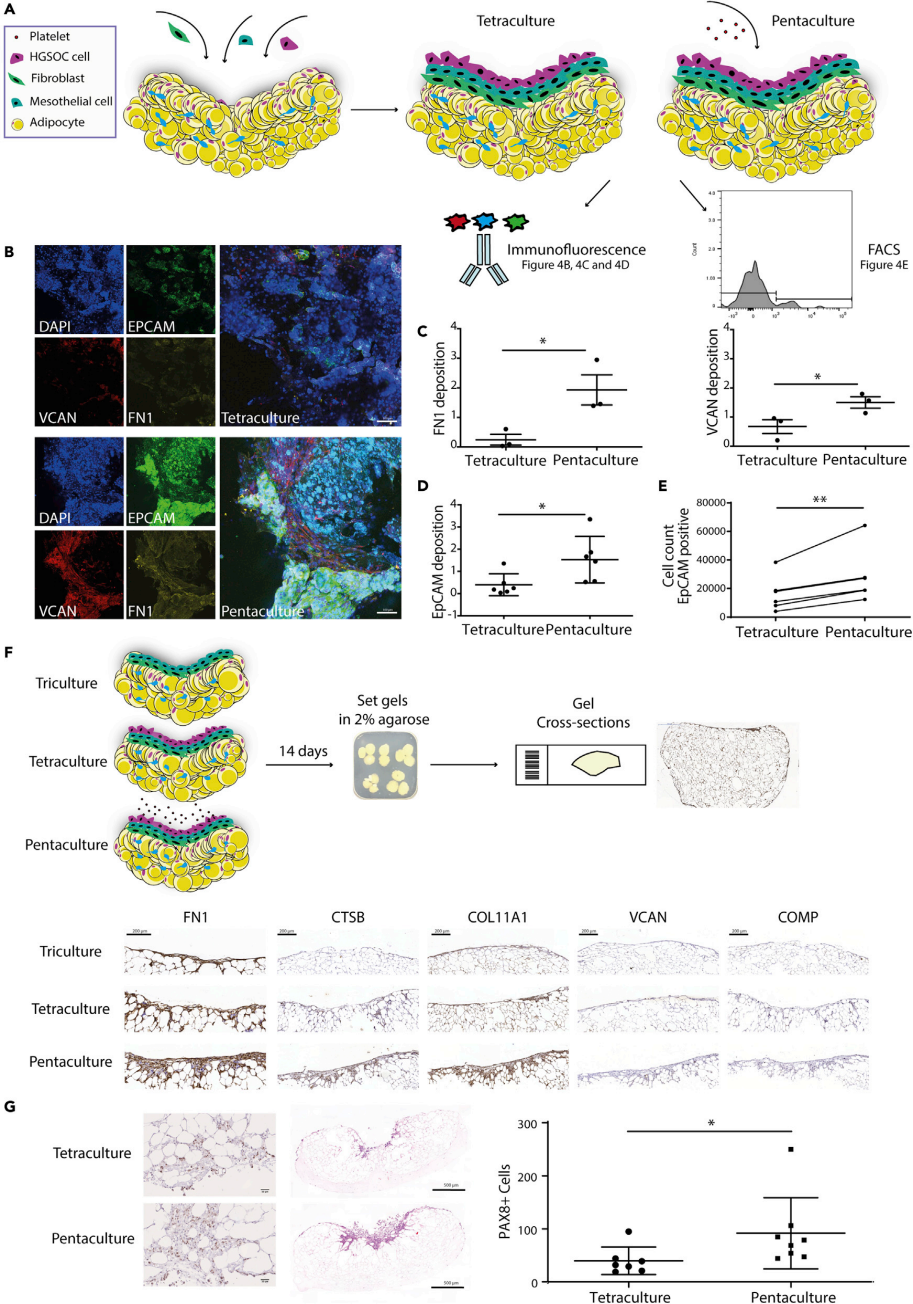
Platelet addition to the tetra-culture model increases ECM deposition and cancer cell invasion (A) Schematic shows the model setup. First, fibroblast cells were plated on top of the adipocyte gel, followed by mesothelial. After 24 hr, HGSOC cells were added on top to form the tetra-culture model. Fresh isolated platelets were then plated on the tetra-culture model to make the penta-culture model. After 7 days, tetra- and penta-culture were analyzed with confocal imaging and FACS. (B and C) (B) IF staining of EpCAM positive G164 cells and FN1 and VCAN in tetra- and penta-cultures (scale bar 100 μm). The increased deposition of FN1 and VCAN in the penta-culture was then quantified in at least 2 gels per condition (n = 3) and plotted (C). (D and E) (D) Quantification of EpCAM deposition demonstrates the presence of a higher number of malignant cells in the penta-culture which is confirmed by flow cytometry analysis on EpCAM positive cells (n = 3) (E). (F) IHC analysis of FN1, CTSB, COL11A1, VCAN, and COMP confirms an increase in ECM deposition in the penta-culture compared to the tetra- and tri-cultures (representative images, n = 3) (scale bar 200 μm). (G) H&E and PAX8 staining of the gels (left panel) shows a greater invasion in the presence of platelets (penta-culture) (scale bar 50-500 μm). PAX8 positive nuclei that were further than 50 μm from the perimeter were counted in tetra- and penta-culture gels respectively (n = 3). Alla data are expressed as mean ± SD (∗p < 0.05 and ∗∗p < 0.01; unpaired t test for 3C and 3D and paired t test for 3E).

To further explore whether platelets were stimulating the production of a poor prognosis ECM, we assessed two of the ECM molecules identified in our previous study on human HGSOC cancer tissue (Pearce et al., 2018) that associated with poor prognosis, namely versican (VCAN), and fibronectin (FN1), within tetra- and penta-cultures (Figure 4A). Using quantitative confocal microscopy, we found that penta-culture models had significantly more VCAN and FN1 deposition within the culture compared to the tetra-cultures, and that this expression was predominantly located where malignant cells were attaching or invading into the model. VCAN and FN1 appeared to be deposited around both malignant cells and stromal cells (likely fibroblasts and/or the mesothelial cells) present in the model (Figures 4B and 4C). We also observed a higher number of EpCAM positive malignant cells in the platelet-treated cultures (Figure 4D) which was confirmed by flow cytometry analysis on the whole cell population isolated from tetra- and penta-cultures (Figure 4E). In addition to an increase in tumor cell numbers in the penta-culture, we had noted that malignant cells tended to form a disorganized corkscrew-like structure on the surface of the model that suggested that the invasion of malignant cells into the tissue model was occurring more readily in the presence of platelets (Video S1). Moreover, the transcriptomic analysis showed in Figure 3 had also identified genes associated with a more invasive phenotype in the penta-cultures.

Video S1. Platelet activated tumour models (pentacultures) have a disorganised malignant and stromal cell organization and evidence of early invasion within penta vs tetra culture.

Further analysis of five MI proteins upregulated in diseased HGSOC, FN1, VCAN, cartilage oligomeric matrix protein, cathepsin B (CTSB), and collagen 11 (COL11A1), revealed an increased expression of all these ECM proteins in the penta-cultures when compared to the tetra-cultures (Figure 4F), which was in keeping with our analysis of the surface of the models (Figures 4A–4E).

### Platelets stimulate malignant cell invasion

As described above (Figure 4F and Video S1), it seemed that platelets may be stimulating invasion of tumor cells within the model. To test this further we stained cross sections of tetra- and penta-cultures with an HGSOC-specific nuclear marker, PAX8, and counted the number of PAX8 positive cells that had invaded into the interior of the model (Figure 4G, left and middle panels). There were significantly more PAX8 positive cells in penta-cultures compared to tetra-cultures (Figure 4G, right panel). Taken together these data indicate that platelets stimulate ECM deposition, tumor cell proliferation, and malignant cell invasion within this human multi-cellular model.

### Platelets stimulate ECM and EMT gene processes in mesothelial cells and malignant cells

**Figure 5:**
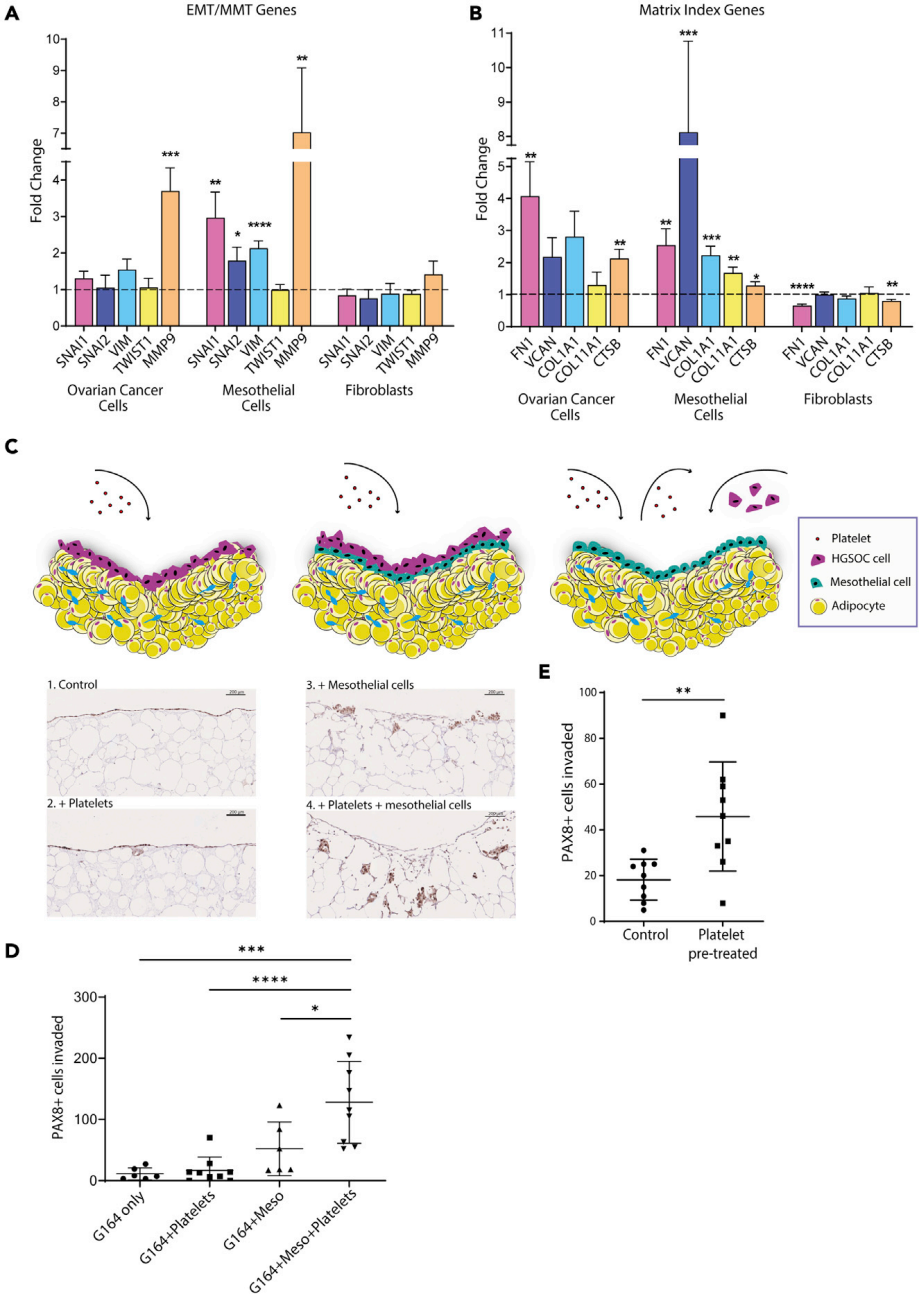
Mesothelial cells are the most affected by platelet activation (A and B) Primary mesothelial cells were co-cultured with 100 platelets/cell for 72 hr before RT-quantitative polymerase-chain reaction analysis of changes in (A) EMT gene expression and (B) Matrix Index gene expression. Y axis shows the relative fold change in gene expression with platelets vs no platelets. (C–E) (C) Schematic figures and PAX8 staining images showing the increased malignant cell invasion upon addition of mesothelial cells and platelets to the model. PAX8 positive nuclei that were further than 50 μm from the perimeter were counted in the different models (n = 3) (scale bar 200 μm) (D) or with/without removing platelets from the culture (n = 3) (E). Data are presented as mean ± SEM. Significance was determined using an unpaired t test or a one-way ANOVA with Tukey's post hoc test where ∗ = p < 0.05, ∗∗ = p < 0.01, ∗∗∗ = p < 0.001, ∗∗∗∗ = p < 0.0001.

We then asked which cell type(s) within the model were facilitating the matrix deposition and tissue invasion phenotype seen in penta-cultures. Using human HGSOC cell lines we observed that the binding between platelets and malignant cells stimulate an EMT phenoyte (Figure 5A) and that platelets bind to the surface of malignant cells generating cell-platelet aggregates (Figure S3A), as previously shown in a murine model (Labelle et al., 2011). Once bound to the malignant cell, platelets become activated (Figure S3A) and are able to alter the chemokine/cytokine profile of our HGSOC malignant cells (Figure S3B and Tables S5 and S6). To further support the finding of increased ECM deposition in the penta-culture model (Figure 4), we analyzed the transcriptomic profile of HGSOC cell lines co-cultured with platelets and we found that platelets stimulate a strong ECM gene profile in malignant cells (Table S6 and Figures S3C–S3G).

Next, we explored whether platelets may be activating invasion and ECM processes through other cells in the model. Using image stream analysis, we compared platelet binding and activation in cultures of primary omental mesothelial cells and primary omental fibroblasts (Figures S4A–S4C) and we found that platelets were activated and bound to both cell types. However, we noted that more platelets were bound and activated on mesothelial cells compared to fibroblasts (Figures S4A–S4C). These data indicate that platelets bind mesothelial cells and become activated in a comparable manner to malignant cells (Figure S3A) but less so with primarily human omental fibroblasts. We then tested whether platelets altered the ECM and EMT gene profile in HGSOC cell lines, primary mesothelial cells, and primary fibroblasts. Platelets increased the expression of EMT and ECM genes in both mesothelial and malignant cells, and the fold change in expression was significantly higher for some genes in mesothelial cells compared to malignant cells (Figures 5A and 5B). This was also confirmed in malignant cells at protein level for two of the EMT genes analyzed (Figure S4D). In contrast, these same EMT and ECM gene profiles did not appear to be altered by platelets in fibroblasts (Figures 5A and 5B). From these data, we concluded that platelets stimulated ECM and EMT gene expression in mesothelial cells and malignant cells but not fibroblasts.

### Platelet activation of mesothelial cells disrupts the mesothelial barrier permitting malignant cell invasion into the tissue

**Figure 6:**
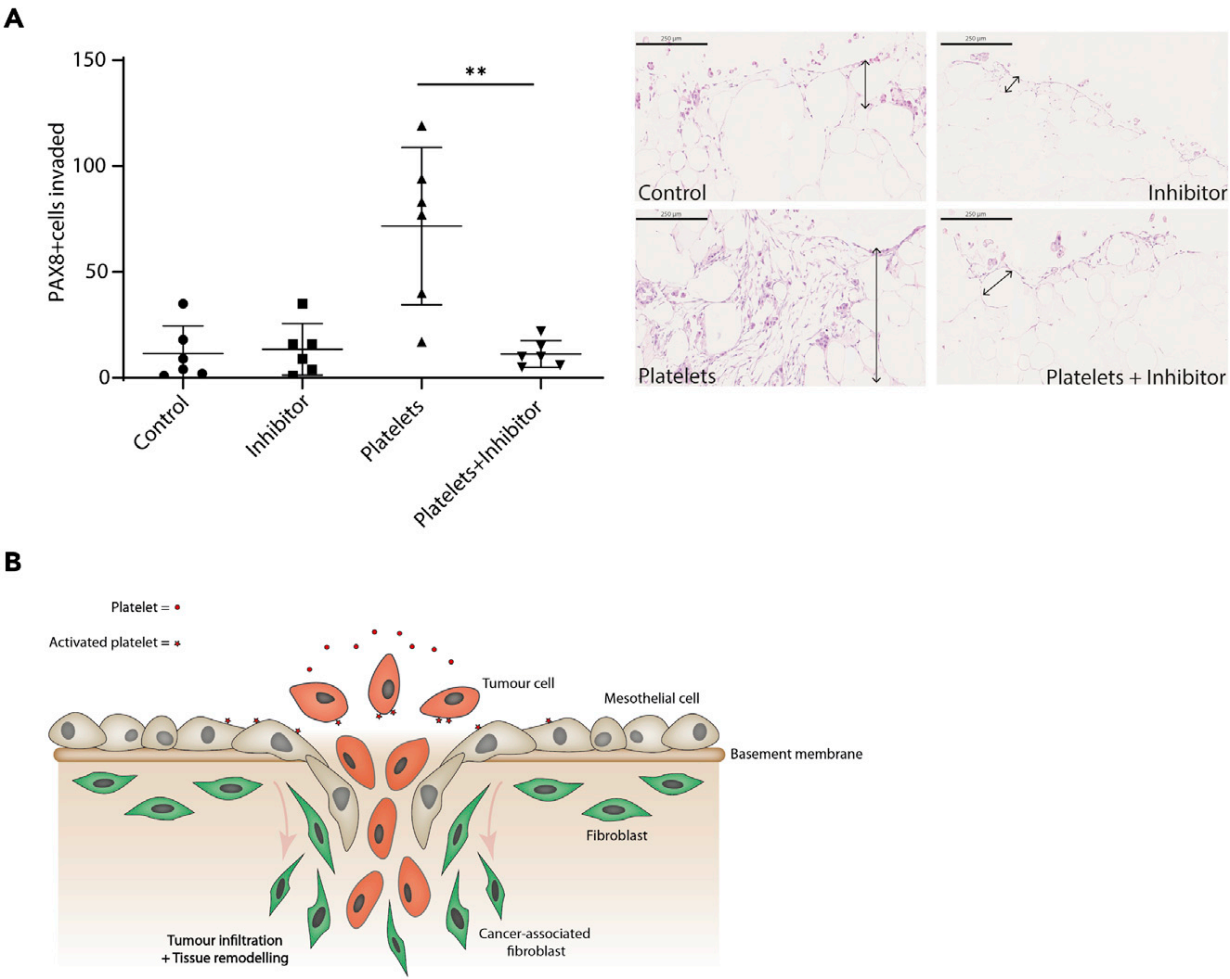
A TGF-inhibitor can revert the invasion phenotype promoted by platelets (A) Mesothelial and G164 cells were plated on top of the adipocyte gels. SB431542, a TGFβ inhibitor, or fresh media was added to the model prior platelets isolation and addition. After 7 days of incubation, gels were fixed and embedded. PAX8 positive nuclei that were further than 50 μm from the perimeter were counted in at least 3 gels for each condition (n = 2) (data are expressed as mean ± SD; one-way ANOVA with Tukey's post hoc test ∗∗ = p < 0.01, scale bar 250 μm). (B) The schematic figure shows the importance of the mesothelial activation by platelets to promote invasion in our model.

We next asked whether platelet activation of malignant cells or mesothelial cells was most important to malignant cell invasion using the multi-cellular model. Since mesothelial cells were also activated by platelets, we decided to investigate whether they were involved in the invasion of malignant cells. Malignant cells were cultured on the adipocyte gels (the base for building the tetra- and penta-cultures described in the previous figures) with or without mesothelial cells and with or without platelets (Figure 5C, left panels, and Figure 5D). We found that malignant cells alone, malignant cells plus platelets, or malignant cells plus mesothelial cells were unable to invade into the tissue model. However, when platelets were added to the co-cultures of malignant and mesothelial cells significant malignant cell invasion occurred (Figure 5C, right panels, and Figure 5D). We next tested whether platelet activation of mesothelial cells alone was enough to support malignant cell invasion. Mesothelial cells were plated on the surface of an adipocyte gel and then activated with platelets prior the addition of malignant cells (Figure 5E). Platelet activation of mesothelial cells was enough to facilitate malignant cell invasion into the tissue, indicating that direct platelet activation of malignant cells was not essential for tissue invasion, if the mesothelial barrier was already activated by platelets. Since we found that the TGFβ pathway was associated with the expression of our ECM signature in HGSOC tissue samples, we wanted to look if the inhibition of this pathway could somehow alter the platelet-driven phenotype we observed in our penta-culture model. To do so, we added a TGFβ inhibitor to the adipocyte gels cultured with mesothelial and malignant cells with or without platelets addition (Figure 6A), and we observed a complete inhibition of malignant cell invasion within the model. Whilst we have not investigated the cellular source of TGFB in this model, it is likely, based on other studies and from our own analysis of the cell lines used here (data not shown), that the primary source of TGFβ is the malignant cells. Taken together, these data demonstrate that platelet activation of mesothelial cells is a requirement for increased tumor cell invasion (Figure 6B), and that it may be more important to target the phenotype in mesothelial cells that platelets stimulate to inhibit malignant cell invasion.

## Discussion

Here, we present a multi-cellular model of early HGSOC metastasis to the omentum that is constructed from primary human cells, relevant early passage HGSOC cell lines and platelets to co-culture up to 5 different cell types. Our approach in generating this multi-cellular model has been to first “deconstruct” the human tissue of interest and then use this as a template and validation for a “reconstruction”. Histopathological comparison of models with the relevant human tissue revealed comparable arrangements of cells indicating the model is partially self-assembling, with the preparation of the 3D primary adipocyte monoculture being the only step requiring physical arrangement of cells by setting adipocytes at high concentration within a collagen 1 matrix. Moreover, there were significant transcriptional similarities in many disease-associated pathways between these models and the diseased tissue.

As recently discussed (Rodrigues et al., 2020), there is a need for 3D multi-cellular models that can recapitulate the complex interactions between malignant cells and their microenvironment. We believe that the approach here, which uses primary adipocytes as the substrate and is based on a deep understanding of the tissue to be modeled, is a useful addition to the field especially in terms of studying cell-cell and cell-ECM interactions. Within the model adipocytes appear to stimulate tumor growth (Figure S5), which occurs *in vivo* in several cancer types that metastasize to fatty tissues including melanoma (Zhang et al., 2018), breast (Wang et al., 2017), and pancreatic cancer (Lengyel et al., 2018). Adipocytes may also be important in stimulating an invasive EMT phenotype in malignant cells (Lengyel et al., 2018). In addition, for the purpose of investigating tissue remodeling, the loose connective tissue structure of adipose tissue provides a clear baseline for visual analysis of tissue remodeling that is occurring during tumor cell invasion that, as shown here, appear as aligned fibers (Figure 4B lower images).

These multi-cellular models were made in a 96-well format and can be used in a semi-high throughput manner, limited only by fresh tissue availability, over approx. 20 days of culture. These models are particularly useful for studying the early stages of metastasis and the tissue remodeling that accompanies this. Whilst we have focused on ovarian omental metastasis, the same approach would lend itself well to other metastatic or primary cancer sites where the normal tissue contains a significant amount of loose connective tissue with a high adipocyte content, such as breast, pancreatic, and liver tissues. As demonstrated here, the model is capable of reproducing platelet-stimulated metastasis and offers the advantage of allowing the dissection of cell-types that are involved in the process. In this context, the model revealed platelet contribution to activation of mesothelial cells in metastasis, which had not been identified from previous *in vivo* studies, where deconvolution of cell roles could not be explored so easily. We did not explore fibroblasts in helping tumor cells to invade within our models because they did not appear to be activated by platelets. However, there is a body of literature that indicates fibroblasts are involved in tumor invasion, for example, fibroblasts lay down ECM tracks that traveling cancer cells follow (for a review that covers this concept please see (Kalluri, 2016)). Additionally, we also explored in this model how platelets stimulate the production of a tumor ECM that has been previously associated with poor prognosis and failure to immunotherapy response. Recently several papers have highlighted the importance of tumor ECM in the response rate to immunotherapy (Chakravarthy et al., 2018; Mariathasan et al., 2018; Tauriello et al., 2018), and there are several clinical trials ongoing which aim to target tumor ECM. Therefore the tissue model presented here may be particularly useful for exploring the role of ECM in tumor immunity and immunotherapy response given the strong matrix signature seen (Figures 3, S2, and S3) and the easily observable and quantifiable changes in ECM remodeling that the model affords (Figures 4A and 4B).

In this work, we used this platform to explore the role of platelets in HGSOC omental metastasis based on the association between platelet processes and our ECM gene signature (Figure 2) found through the analysis of human cancer tissue (Pearce et al., 2018). Platelets have a long association with disease progression and metastasis. Classical studies indicate a role for them in shielding circulating tumor cells from immunosurveillance and potentially aiding tumor cells in hijacking the process of leukocyte rolling during metastasis (Laubli and Borsig, 2010). However, more recent studies have found that platelets may also be important in establishment of two separate stages in disease progression, namely the establishment of the early metastatic niche (Haemmerle et al., 2017; Labelle et al., 2011, 2014) and secondly the vascularization and proliferation of established tumors (Haemmerle et al., 2016; Hu et al., 2017). Adding to this, the work presented here provides a fourth role for platelets in cancer dissemination and progression through activation of the tissue mesothelial surface and the deposition of a tumor promoting and therapy inhibiting extracellular matrix. This tumor-associated extracellular matrix appears to be driven through TGFβ and Hedgehog signaling, as we found using a multi-cellular tri-culture HGSOC model, built using the same principles described here (Delaine-Smith et al., 2021).

Evidence from epidemiological studies of long-term regular aspirin use, an inhibitor of platelet activation, is associated with reduced cancer risk including ovarian cancer, further implicating a role for platelets in this disease (Cuzick, 2017). Therefore, taken together, cancer therapies that target platelet activation or block cell-platelet binding may inhibit metastasis, tumor growth, and the development and maintenance of TMEs that are therapy resistant. Targeting platelets in combination with emerging and established cancer therapies may be a promising avenue of cancer therapy, and we suggest that the model presented here is an ideal test platform for these studies and others where tissue invasion and ECM remodeling is involved.

### Limitations of the study

Other cell types have also been implicated in the establishment of the metatstatic niche, in particular granulocytes and macrophages, which are not included within the model presented here.

## STAR★Methods

### Key resources table

**Table:** 

REAGENT or RESOURCE	SOURCE	IDENTIFIER
**Antibodies**		

Monoclonal Mouse Anti Alpha Smooth Muscle Actin	Sigma	Cat #A2547; RRID:AB_476701
Polyclonal Rabbit Anti Versican	Sigma	Cat# HPA004726, RRID:AB_1080561
Polyclonal Rabbit Anti COL11A1	Sigma	Cat# HPA052246; RRID:N/A
Polyclonal Rabbit Anti FN1	Sigma	Cat# F3648, RRID:AB_476976
PE anti-human CD42b	Biolegend	Cat# 303905, RRID:AB_314385
Monoclonal Rabbit anti-fibroblast activation protein, alpha	Abcam	Cat# ab207178, RRID:AB_2864720
Monoclonal Rat anti COMP	Abcam	Cat# ab11056, RRID:AB_297708
Monoclonal Mouse anti CTSB	Abcam	Cat# ab58802, RRID:AB_940824
Polyclonal Rabbit anti PAX8	Novus	Cat# NBP1-32440, RRID:AB_2283498
Human anti EpCAM Alexa Flour 488	ThermoFisher	Cat# 53-8326-41, RRID:AB_11220074
APC anti-human CD62P (P-Selectin)	Biolegend	Cat# 304910, RRID:AB_314482
Human Fibroblast Activation Protein alpha PE-conjugated Antibody (FAP-PE) (Clone # 427819)	R&D systems	Cat# FAB3715P; RRID: N/A
Human alpha-Smooth Muscle Actin APC-conjugated Antibody (αSMA-APC) (Clone #1A4)	R&D systems	Cat# IC1420A, RRID:AB_10890600
Human anti EpCAM Brilliant Violet 650	Biolegend	Cat# 324225, RRID:AB_2562734
Biotinylated goat anti-rabbit IgG antibody 1.5mg	Vector Labs	Cat# BA-1000, RRID:AB_2313606
Biotinylated goat anti-rabbit IgG antibody 1.5mg	Vector Labs	Cat# BA-9200, RRID:AB_2336171
Polyclonal Rabbit anti CALB2	Sigma	Cat# HPA007305, RRID:AB_1078386

**Chemicals, Peptides, and Recombinant Proteins**		

Trypsin-EDTA solution 10X	Sigma	T4174
DMEM/F12 with Glutamax	Thermo Fisher Scientific	31331093
FBS	Fisher Scientific	10500-064
Collagenase type I powder	Thermo Fisher Scientific	17100017
SB431542 hydrate	Sigma	S4317
EDTA	Thermo Fisher Scientific	AM9262
L-Ascorbic acid 2-phosphate sesquimagnesium salt hydrate	Sigma	A8960
Collagen I from rat tail	Thermo Fisher Scientific	A1048301
DMEM low glucose 10x	Sigma	D2429
Agarose, low gelling temperature	Sigma	A0701
Goat serum 100ml	Life Technologies	16210064
Fixable Viability Dye eFluor 450	eBioscience	65-0863-18
Agilent RNA 6000 Pico Reagents	Agilent	5067-1514
Medium-199	Thermo Fisher Scientific	22350029
Fluorescein diacetate	Sigma	F7378-5G
Ethidium Homodimer I Solution	Sigma	E1903
Insulin-Transferrin-Selenium-Sodium Pyruvate (ITS-A) (100X)	Thermo Fisher Scientific	51300044
Zytomed Antibody diluent	Bioscience LifeSciences	ZUC025-500
Bovine Serum Albumin	Sigma	A4503
Hydrogen Peroxide 30% (w/v) (100 Volumes), Extra Pure SLR, Fisher Chemical	Fisher Scientific	10687022
Vectastain Elite ABC HRP Kit	Vector Laboratories	PK-6100
SIGMAFAST DAB Tablets	Sigma	D4293
Hematoxylin Solution, Gill No. 1	Sigma	GHS116
Formalin solution neutral buffered 10	Sigma	HT501128-4L
DPX Mountant for histology	Sigma	06522
Triton X-100	Sigma	T8787
DAPI	Biotium	40043
Sodium Chloride	Sigma	71383
Potassium Chloride	Sigma	P9541
Sodium bicarbonate	Sigma	S5761
HEPES	Sigma	H0887
Magnesium chloride	Sigma	M8266
Sodium phosphate dibasic heptahydrate	Sigma	S2429
Prostaglandin E1	Sigma	P5515
Apyrase	Sigma	A6535

**Critical Commercial Assays**		

RNeasy Mini Kit (50)	Qiagen	74104
High-Capacity cDNA Reverse Transcription Kit (200 reactions)	Thermo Fisher Scientific	4368814
iTaq™ Universal Probes Supermix (10 x 1ml)	Biorad	1725132

**Deposited Data**		

RNASeq platelets on G164-MCMs	GEO	GSE155547
RNASeq platelets on G164-spheroids	GEO	GSE155546

**Experimental Models: Cell Lines**		

Human AOCS1	Kindly gifted by Prof D Bowtell's lab	(Tamura et al., 2020)
Human G164	isolated in our lab	(Tamura et al., 2020)

**Oligonucleotides**		

	See Table S7	N/A

**Software and Algorithms**		

FlowJo 9.4.6	Treestar Inc.	https://www.flowjo.com/
GraphPad Prism 8.3.0	GraphPad	https://www.graphpad.com/scientific-software/prism/
R 3.1.3	NA	http://www.R-project.org
HTSeq	https://htseq.readthedocs.io/en/release_0.11.1/	Anders et al., 2014
EdgeR	Bioconductor	Robinson and Oshlack, 2010
Limma	Bioconductor	Ritchie et al., 2015
GSEA	https://www.genepattern.org/	Subramanian et al., 2005
Biorender	https://biorender.com/	Some graphical abstract components were created with Biorender.com

### Resource availability

#### Lead contact

Further information and requests for resources and data should be directed to the lead contact, Oliver Pearce (o.pearce@qmul.ac.uk), Barts Cancer Institute, Queen Mary University of London Charterhouse Square EC1M 6BQ, London, UK.

#### Materials availability

This study did not generate new unique reagents.

#### Data and code availability

The accession number for the RNASeq data reported in this paper is Gene Expression Omnibus (GEO): GSE155547 (platelets on G164-MCMs) and GSE155546 (platelets on G164-spheroids).

### Experimental models and subject details

#### Isolation and of mesothelial cells, fibroblasts, and adipocytes from human omentum

Samples were collected by Barts Gynae Tissue Bank (HTA license number 12199. REC no: 10/H0304/14) from women undergoing omentectomy and omental biopsy after giving written informed consent (age range 40-87 yo). Use of the tissue for medical research was approved by a UK national review board. Approximately 10 cm^3^ of omentum was submerged in 0.25% trypsin (Sigma-Aldrich) and incubated at 37°C for 20 min. Trypsin was neutralized with growth medium (DMEM:F12 (Gibco) with 10% heat-inactivated FBS (Hyclone) and penicillin-streptomycin (Sigma-Aldrich)). The cell suspension was centrifuged at 200 x *g* for 5 min and the pelleted mesothelial cells resuspended in growth medium and cultured at 37°C, 5% CO_2_. After isolation, cells were grown to confluency. The cells were defined as mesothelial cells based on the cobblestone morphology and flow cytometry for calretinin positivity and EpCAM negativity. We estimate purity to be >95%. The tissue was then minced with scalpels and suspended in 0.5 mg/ml collagenase type I (Gibco) in growth medium and placed in a shaking incubator at 50 rpm, 37°C for 75 min. Digested tissue was passed once through a 250 μm tissue strainer (Thermo-Fisher) and the adipocyte layer collected and washed by centrifuging twice at 200 x g in DMEM with 5% FBS. The fibroblast pellet from the first adipocyte wash was resuspended in growth medium and cultured at 37°C, 5% CO_2 ._ After three days, any unattached cells were washed away with 1X PBS and the morphology of the attached cells was checked and then referred to as fibroblasts. Multiple fibroblast donors have been used for each experiment. Cells were defined as fibroblasts based on their spindle like morphology and by flow cytometry (calretinin- EpCAM-) and by IF for SMA+FAP+. We estimate purity to be >95 %.

#### Ascite and peritoneal wash fluids collection and processing

Uncoagulated ascite and peritoneal washes fluids were collected by Barts Gynae Tissue Bank from women undergoing ovarian cancer surgery. Ascite fluids were spun down at 1000 x g for 10 min to sediment platelets. Platelets from peritoneal washes were isolated following the isolation of platelets from peripheral blood samples described below. After the latest wash in MTH buffer, platelets from peripheral blood and peritoneal washes were resuspended in FACS buffer prior the FACS staining. Isolated platelets were counted and stained for CD42b to identify the platelet population and for CD62P to identify the activated platelets.

#### Isolation of platelets from peripheral blood samples

Blood was collected from female healthy donors under the permissions granted in the Barts Gynae Tissue Bank HTA license (number 12199. REC no: 10/H0304/14) and centrifuged at 180 x g for 15 min with no brake. The supernatant was removed and prostaglandin E1 and apyrase were added to a final concentration of 1mM and 100 U/ml respectively. The platelet suspension was centrifuged at 100 x g for 4 min and the supernatant centrifuged at 1000 x g for 10 min and resuspended in 5 ml MTH buffer (134 mM NaCl, 2.9 mM KCl, 0.34 mM Na_2_HPO_4_, 12 mM NaHCO3, 20 mM HEPES, 1 mM MgCl2, 0.1% glucose, 0.35% BSA, pH 7.4) before addition of PGE1 and apyrase as previously. The platelet pellet was washed once more in MTH buffer (with PGE1 and apyrase) and resuspended in growth medium for co-culture experiments. After isolation, platelet purity was checked by viewing using a cytometer which revealed no cell contamination and flow cytometry using size, granularity, and platelet marker CD42. Purity was calculated as >99 %.

### Method details

#### Casting adipocytes into collagen gels

Purified adipocytes were combined with the following reagents to give 1 mg/ml collagen adipocyte gels: 1ml of adipocyte suspension, 1 ml of 3 mg/ml rat-tail collagen I (Gibco), 100 μl of 10x DMEM low-glucose (Sigma), 48 μl of 1M NaOH and 852 μl H_2_O. The adipocyte-collagen mixture is prepared on ice, quickly transferred in 100 μl aliquots in a 96 well dish and incubated at 37^o^C, 5% CO_2_ for 30 min. Gels were then gently transferred to 24 well dish, cultured in growth medium and used for experiments with other cells within 7 days from the isolation. Adipocytes were characterized by morphological features of size and shape. Once set in a collagen gel they were stained for perilipin-1 expressed by mature adipocytes. Based on these two analysis no other cell contamination was detectable.

#### Tri-, tetra- and penta- culture models set up

Adipocyte gels were anchored to the bottom of a 96 well dish by incubating them on top of 20 μl of 1 mg/ml rat-tail collagen I solution (per 100 μl of 3 mg/ml collagen: 30 μl DMEM low-glucose 10x, 4.8 μl of 1M NaOH, 165 μl H_2_O) for 10 min. Gels were then seeded with 40,000 primary human omental fibroblasts per gel and incubated for 2 hr. The medium was then replaced with 200 μl of mesothelial cell medium (Medium 199 (Gibco) with insulin-transferrin-selenium (Gibco), 10% heat-inactivated FBS (Hyclone) and penicillin-streptomycin (Sigma-Aldric)) containing 200,000 human primary mesothelial cells and incubated for 24 hr. G164 or AOCS1 ovarian cancer cells (Tamura et al., 2020) were then seeded at 40,000 cells per gel and incubated for 24 hr before loosening and transferring the gels to a 24 well dish in growth medium. To make up the penta-culture model, a ratio of 1:100 cancer cells:isolated platelets was added to the culture. After 24 hrs the media was replaced in all wells and SB431542 (TGFbeta inhibitor) and/or 4 million platelets/gel added. The medium was replaced every 3-4 days until endpoint. AOCS1 (PMID: 25670170) and G164 cell lines were established from HGSOC omental metastasis tumors and subjected to STR sequencing analysis. AOCS1 cells were maintained in RPMI1640 with 10% FBS and 1% L-Glutamine and 1% penicillin-streptomycin. G164 cells were maintained in DMEM:F12 plus Glutamax supplemented with 4% human serum (Sigma Aldrich) and 1% penicillin-streptomycin.

#### Flow cytometry staining

Co-culture gels were digested in 1 mg/ml collagenase type I (Thermo-Fisher) in serum-free DMEM for 1 hr at 110 rpm, 37°C. Gels were then disaggregated and 0.5% trypsin-EDTA (Sigma-Aldrich) was added before incubation in a water bath at 37°C for 30 min. DMEM with 10% FBS was then added 1:1 to the cell suspension and centrifuged at 200 x g for 5 min. The pellet was resuspended in 250 μl of PBS and the cells counted using a hemocytometer. Cells were washed in PBS and resuspended in 100 μl of FACS buffer (1X PBS + 2 mM EDTA and 2% FBS) containing 2 μg/ml of primary antibody (see antibody table) for 30 min. After two washes in FACS buffer cells were fixed with a 1:1 mixture of neutral buffered formalin (Sigma-Aldrich) and FACS buffer for 10 minutes on ice. Cells were stored in the dark at 4°C before analysis by flow cytometer. A minimum of 10,000 events were acquired on a BD LSRFortessa flow cytometer (BD Biosciences) using BD FACSDiva software (BD Biosciences) and analyzed using FloJo v10.6.1 software.

#### Live/dead assay

Gels were immersed in PBS containing 20 μg of fluorescein diacetate (Sigma-Aldrich) and 4 μM ethidium homodimer (Sigma-Aldrich) for 10 min at 37°C, after which they were placed on a glass slide. Gels were imaged using a Zeiss LSM610 confocal microscope.

#### Immunohistochemistry

Formalin-fixed paraffin-embedded sections (4 μm) of omentum samples or co-cultures gels were heated for 1 hr at 60°C and then submerged twice in xylene for 5 min. Immunohistochemistry was performed using a Vectastain ABC Kit (Elite), as per the manufacturer's instructions. Slides were rehydrated and heated-antigen retrieval was performed using citric acid-based antigen unmasking solution (Vector Laboratories) and heated in a 2100 antigen-retriever (Aptum Biologics). Endogenous peroxidase activity was blocked using 3% H2O2 in PBS and sections were then blocked with 5% BSA. The primary antibody was added in antibody diluent (Zytomed) and covered at room temperature for 1 hr. Slides were incubated with a biotinylated secondary antibody (Vector) for 30 minutes. Subsequent steps were carried out according to the protocol included with the Vectastain Elite ABC HRP kit, after which the slides were incubated with DAB solution made using Sigmafast DAB tablets (Sigma-Aldrich). Finally, slides were counterstained in 50% Gill’s hematoxylin I, washed with water and dehydrated. After leaving to dry, coverslips were affixed using a drop of DPX mountant (Sigma-Aldrich).

#### Quantification of cancer cell invasion

Adipocyte co-culture gels were fixed in 10% neutral buffered formalin then cast in 2% agarose gels, vertically cross-sectioned and re-cast into 2% agarose before dehydration and paraffin embedding. Sections were stained as described in the immunohistochemistry protocol for PAX8 and the slides were scanned using a NanoZoomer S210 (Hamamatsu). For each gel section the number of PAX8 positive nuclei in the interior of the gel that were further than 50 μm from the perimeter was counted.

#### 2D platelet co-culture

Cells were plated into a 6-well dish at 200,000 cells/well in growth medium. After 24 hr incubation, platelets from a healthy donor were isolated and washed as described above and 100 platelets/seeded cells were added to one well, media only was added to the remaining wells. Total RNA was collected after a 72 hr incubation.

#### RNA isolation and real-time quantitative PCR

Total RNA was prepared from cell cultures using the RNeasy Mini Kit and the RNase free DNase set (Qiagen). After washing cells or the tetra- and penta-cultures gels in PBS and addition of 350 μl of RLT buffer, RNA was isolated according to the manufacturer’s instructions. The RNA was quantified using the NanoDrop 2000c (Thermo-Fisher Scientific). The reverse-transcription reaction was carried out using the T100 Thermal Cycler (Bio-Rad) and the High-Capacity cDNA Reverse Transcription Kit (Applied Biosystems) according to the manufacturer’s instructions. The PCR reaction was carried out using (Applied Biosystems) and FAM-MGB labeled Taqman gene expression assay probes. The reaction mixture for each replicate contained 5 μl of cDNA at 1 ng/μl, 10 μl of Supermix, 1 μl of probe, 4 μl of RNase-free water, and was run on a StepOnePlus Real-Time PCR System (Applied Biosystems). VIC labeled 18s RNA was used as a housekeeping gene. The delta-delta Ct calculation has been used to quantify the gene expression. Results are shown as Log_2_ (fold change) of the Rq value.

#### ICGC analysis based on matrix index

The ICGC_OV read counts across 93 primary tumors were extracted from the exp_seq.OV-AU.tsv.gz file in the ICGC data repository Release 20 (http://dcc.icgc.org). Overall survival (OS) was extracted from the donor.OV-AU.tsv.gz file. Matrix index was calculated for each sample survival analysis was performed as described in (Pearce et al., 2018). Differential expression of the 72 matrix index high versus 21 matrix index low samples was undertaken using R package limma (Ritchie et al., 2015). GSEA analysis was run using ranked t-statistic of all genes for MSigDB canonical pathways (Subramanian et al., 2005).

#### RNA-seq and analysis

RNA-seq was performed by the Wellcome Trust Centre for Human Genetics (Oxford, UK) to approximately 30x mean depth for the G164 spheroids or approximately 60x for the MCM cultures. The sequencing was carried out on the Illumina HiSeq4000 strand-specific, generating 150bp paired-end reads, strand-specific. RNA-Seq reads were mapped to the human genome (hg19, Genome Reference Consortium GRCh37) in strand-specific mode as part of the Wellcome Trust Centre pipeline. Number of reads aligned to the exonic region of each gene were counted using htseq-count based on the Ensembl annotation (Anders et al., 2014). Only genes that achieved at least one read count per million reads (cpm) in at least twenty-five percent of the samples were kept. Conditional quantile normalization was performed counting for gene length and GC content and a log_2_ transformed RPKM expression matrix was generated. Differential expression analysis was performed using R packages EdgeR and limma (Robinson and Oshlack, 2010). GSEA analysis was run using ranked t-statistics of all genes from the comparison Penta-culture vs Tetra-culture for MSigDB Canonical pathways. Significantly DE genes (FDR < 0.05) from the comparison G164 Spheroids plus Platelets vs Spheroids were ranked by log_2_ fold change used for GSEA for MSigDB Canonical pathways. RNA-Seq data have been deposited in Gene Expression Omnibus (GEO) under the accession numbers GSE155547 (platelets on G164-MCMs) and GSE155546 (platelets on G164-spheroids).

#### Immunofluorescence of 3D cultures

Gels were fixed in 10% neutral buffered formalin. Subsequently, Triton X-100 (0.5% in PBS) was added to the gels and incubated for 10 min. Gels were washed and incubated in blocking solution (PBS with 5% BSA), which was replaced with the primary antibody in antibody diluent (Zytomed) and incubated overnight at 4°C. Gels were then washed before a fluorescent secondary antibody was added in antibody diluent and protected from light while shaking for 1 hr. Finally, gels were incubated with PBS containing 0.4 μg/ml DAPI, washed in PBS for 5 min, and imaged using a Zeiss LSM510 confocal microscope using a 10x or 20x air objective. Tetra- and penta- culture images were acquired using a field of view equal to 238.1 x 238.1μm containing 1024x1024 pixels. All imaging conditions were kept constant for all the experiments.

#### Image stream analysis from 2D co-culture

500,000 cells and/or platelets were scraped in PBS and pelleted by centrifugation at 1000 x g for 5 minutes and resuspended in FACS buffer (2mM EDTA, 2% FBS in PBS) on ice. Normal protocol for FACS staining was followed as described previously. A minimum of 1,000 events was acquired on an Amnis ImageStream X Mk II (Luminex) using IDEAS v6.2 for both acquisition and analysis.

### Quantification and statistical analysis

Statistical analyses were performed in GraphPad Prism or the programming language R (version 3.1.3). To compare two groups, unpaired t-test was used while for pairwise comparisons a two-way paired t-test was used. To compare more than two groups, one-way ANOVA with Tukey’s HSD test were used if not otherwise indicates. The differential expression analysis was performed in Edge R using limma (PMID:25605792) while gene set enrichment analysis (GSEA) was completed using the GSEA software (Mootha et al., 2003) to identify the canonical pathways gene sets from the Molecular Signatures Database (MSigDB-C2-CP v6.2). Data were considered statistically significant from *p* < 0.05.

## Supplemental information

Document S1. Figures S1–S5

Table S1. Analysis of DE genes from HGSOC patient tissues split by high vs low MI signatures, Related to Figure 2 Data from

Table S2. DE genes from RNASequencing of penta-vs tetra-cultures, Related to Figure 3

Table S3. Matrisome filtered DE genes from RNASequencing of penta-vs tetra-cultures, Related to Figure 3

Table S4. Pathway analysis of RNASequencing of penta-vs tetra-cultures. (A) GSEA, and (B) Gene ontology, Related to Figure 3

Table S5. Chemokines are released by G164 and AOCS1 spheroids, Related to Figure 4 Spheroids of G164 and AOCS1 with or without platelets were set in low adherence plates. Spheroids were then lys ed, RNA was extracted and RT-qPCR was carried out to evaluate the chemokine release. Corrected Rq values showing up- and down- regulation of a panel chemokines in G164 and in AOCS1.

Table S6. Analysis of DE genes from G164 spheroids with platelets vs G164 spheroids alone, Related to Figure 4 Table 1. list of differentially expressed genes. Table 2 GSEA analysis

Table S7. Oligonucleoutide table, Related to STAR Methods
