# Supplementary material for: A human multi-cellular model shows how platelets drive production of diseased extracellular matrix and tissue invasion
Source: iScience. 2021 May 29;24(6):102676. doi: 10.1016/j.isci.2021.102676 (PMC8215303; doi:10.1016/j.isci.2021.102676)
Supplement: Document S1. Figures S1–S5 [file mmc1.pdf]

iScience, Volume *24*

Supplemental information

A human multi-cellular model shows how platelets drive production of diseased extracellular matrix and tissue invasion

Beatrice Malacrida, Sam Nichols, Eleni Maniati, Roanne Jones, Robin Delanie-Smith, Reza Roozitalab, Eleanor J. Tyler, Morgan Thomas, Gina Boot, Jonas Mackerodt, Michelle Lockley, Martin M. Knight, Frances R. Balkwill, and Oliver M.T. Pearce

A

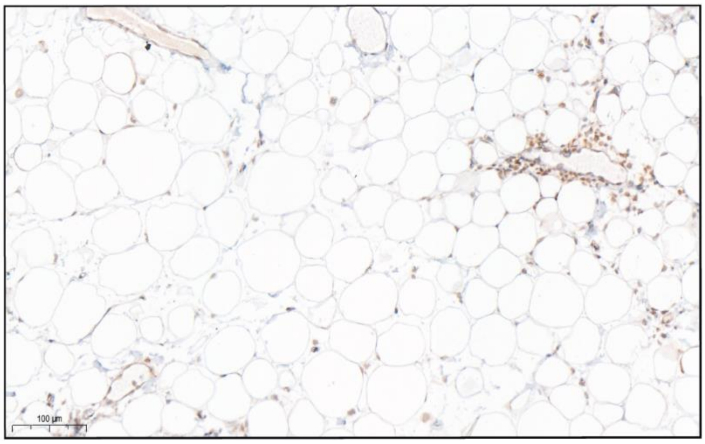

Supplemental Figure 1. MCMs mimic human omental tissue. Related to Fig 1. (A) Human omental metastatic tissue stained for PAX8 to highlight the malignant cell area (scale bar 100 µm).

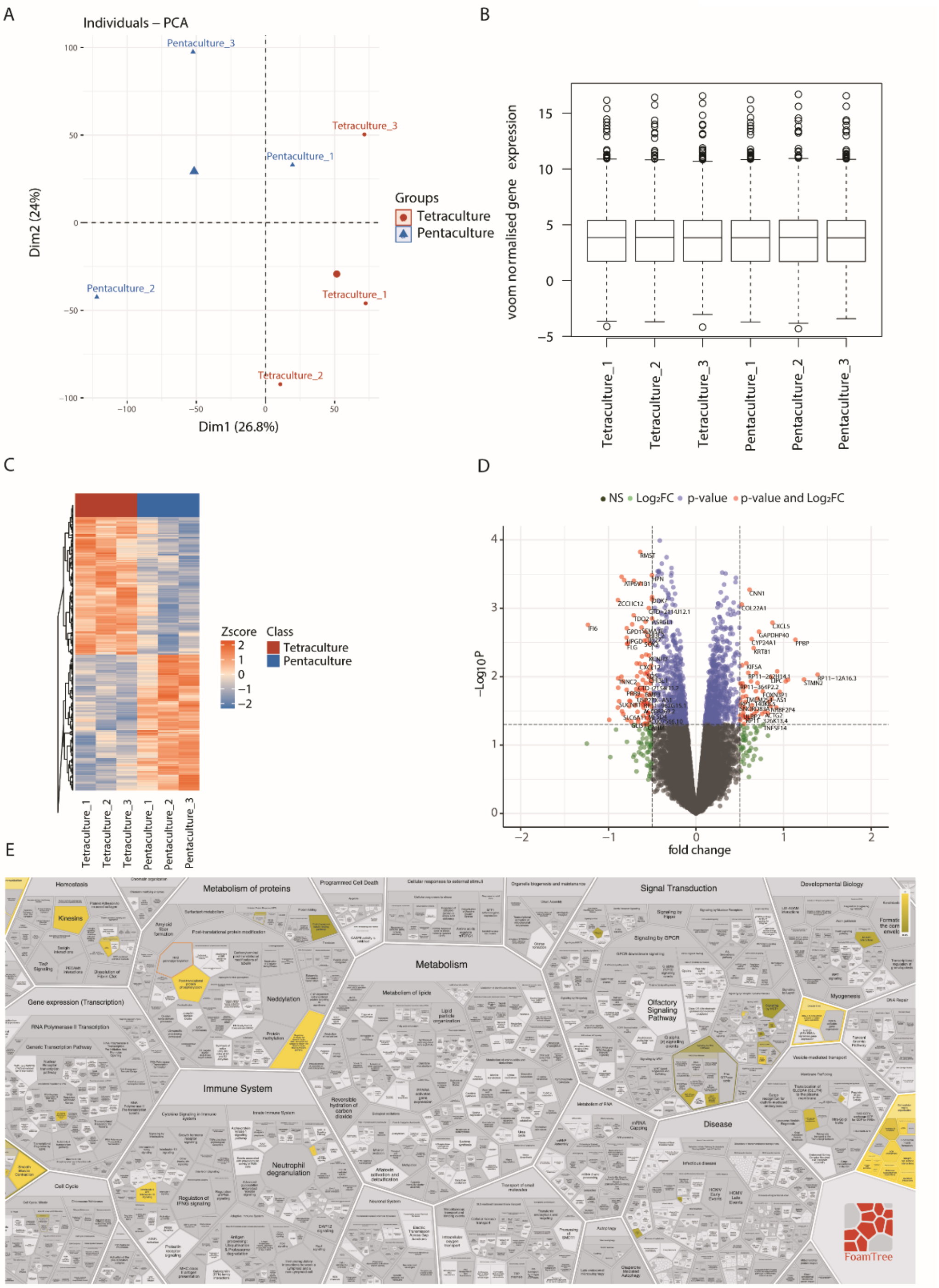

Supplemental Figure 2. Transcriptomic analysis of penta- vs tetra- cultures. Related to Fig. 3 (**A**) PCA analysis comparing experimental replicates (n=3 per group) between tetra- (red) vs penta- cultures (blue) showing a separation between the two models. **(B)** Voom normalized gene expression across all tetra- and penta- culture samples (n=6) shows similar expression distribution. **(C)** Heatmap of DE genes between penta- and tetra- culture samples. **(D)** Volcano plot of penta- vs tetra-culture genes. Gene expression highlighted in red are significantly altered based on a $p < 0.05$, and fold change of 2. **(E)** Reacfoam analysis of DE genes from panel D reveals significant enrichment of ECM organisation processes in penta-cultures. DE expressed genes between penta- vs tetra- cultures were analysed using reactome.org to identify pathway enrichment. Yellow pathways = entities pathway pvalue<0.05. Brighter yellow = smaller pvalue.

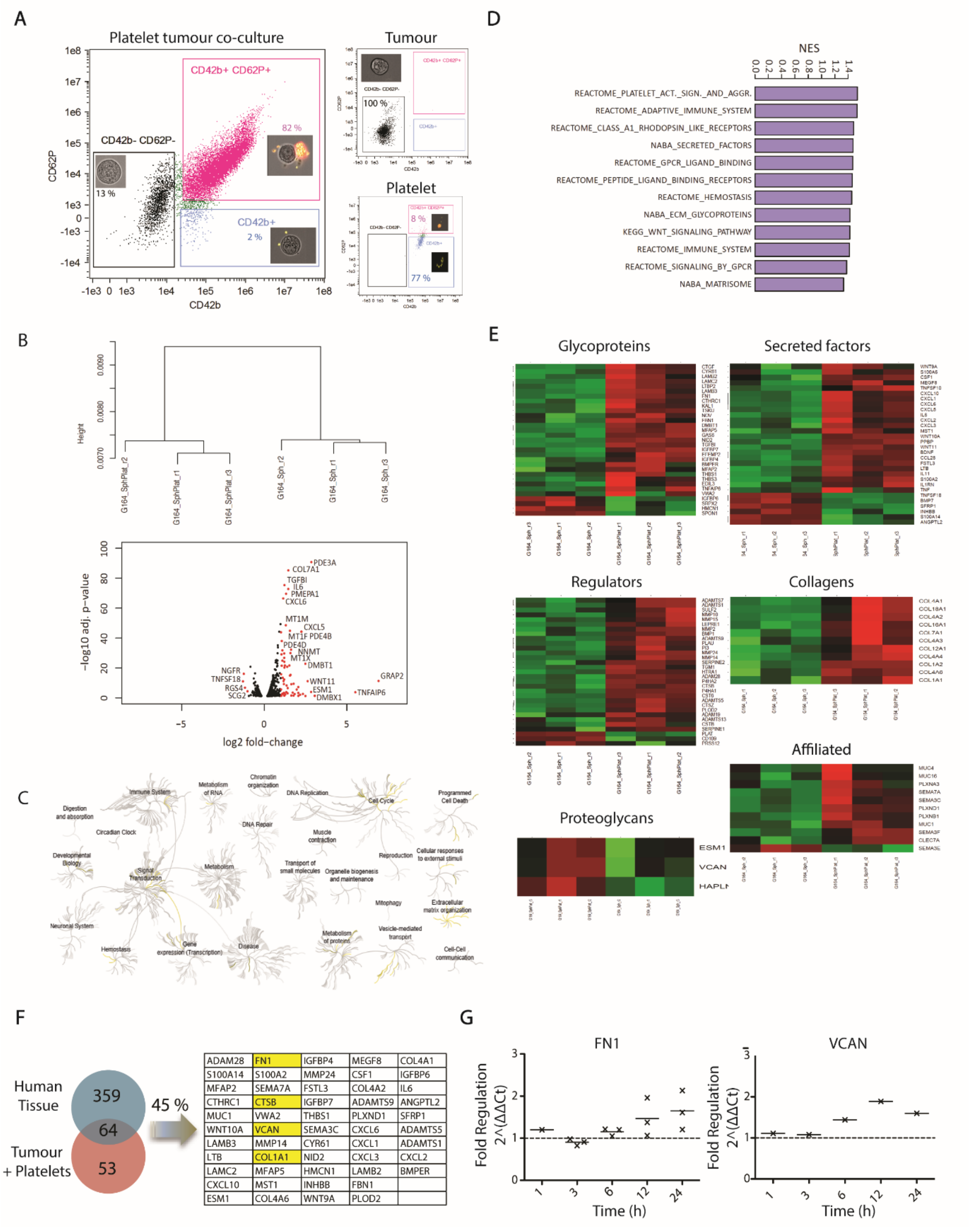

Supplemental Figure 3. Platelets bind to malignant cells and stimulate ECM deposition. Related to Fig. 5. (A) Image stream analysis demonstrate that platelets (CD42b^{+}) become activated (CD62P^{+}) once they form aggregates with HGSOC tumour cells (n=2). **(B)** RNAsequencing analysis of HGSOC cell line G164 in co-culture with platelets or alone. Hierarchical cluster analysis (top panel) reveals samples split based on the presence of platelets in the culture, and the volcano plot illustrating DE genes

(bottom panel) in spheroids with platelets vs speroids alone are mostly associated with a change in cell secretome.Red dots correspond to genes with $log_2FC > |1|$ and adj. p-value < 0.05 **(C)** Rectome.org analysis of DE genes highlights platelets are mostly altering ECM pathways in malignant cells, also seen in **(D)** pathway analysis of DE genes from G164 with platelets vs G164 alone are mostly associated with ECM remodelling, immunity (probably associated though ECM remodelling), in addition to the expected platelet activation process. Barplot illustrates normalised enrichment scores (NES) of top signignificantly changing canonical pathways ($p < 0.05$). **(E)** Heatmaps of DE genes from G164 with platelets vs G164 alone (adj. p-value < 0.05) show all of the main matrisome classes are represented. **(F)** Comparison of matrisome only DE genes from our previous human tissue analysis[5] with matrisome only DE genes from G164 with platelets vs G164 alone reveals a 45 % overlap including 4 (highlighted yellow) of 6 matrisome genes we found upregulated in tumour matrisomes associated with poor prognosis in HGSOC[5].

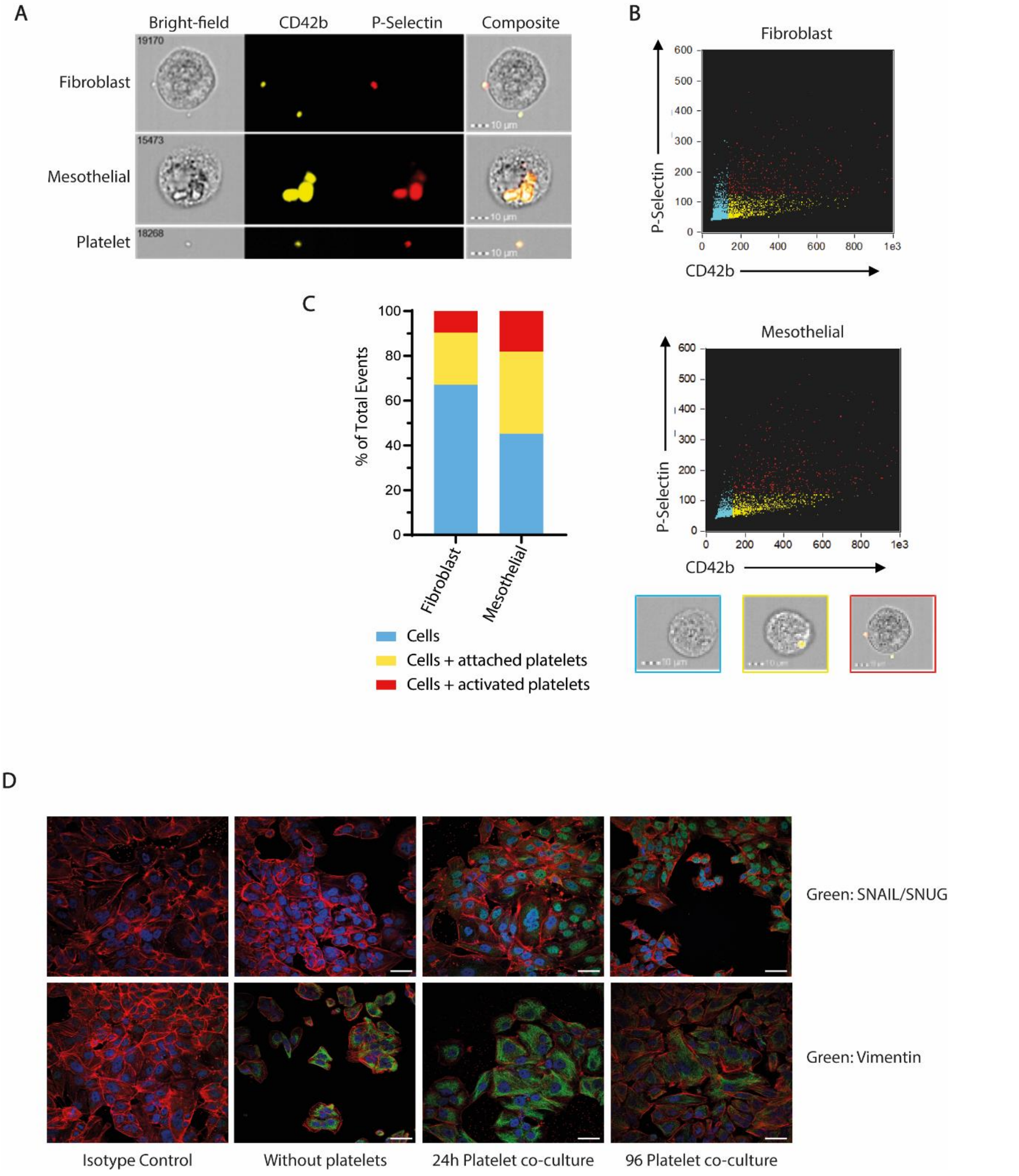

Supplemental Figure 4. Platelets bind mesothelial cells with greater affinity than fibroblasts. **Related to Fig. 5.** Primary fibroblasts or mesothelial cells were co-cultured with platelets and analysed using imagestream for platelet binding and platelet activation. **(A)** Representative bright-field, CD42b fluorescence, P-selectin fluorescence and composite images of one event. **(B)** Scatterplot of CD42b plotted against P-Selectin for each cell type. Blue dots: cells without bound platelets, yellow dots: cells with ≥1 bound platelet, red dots: cells with ≥1 bound and activated platelet (exemplified by accompanying composite images). **(C)** Quantification of scatterplots represented as a percentage of total events, and right panel, example of a scatter plot collected for mesothelial cells. The P-Selectin positive events are presented as a percentage of CD42b positive events. Scale bars: 10 µm. **(D)** AOCS1 were co-culture in presence of platelets for 24 or 96h. IF staining shows an increase in both SNAIL/SNUG (upper panels) and vimentin (lower panels). Red = phalloidin, Green = SNAIL/SNUG (upper panels) and vimentint (lower panel), Blue = DAPI (n=2)(scale bar 200 µm).

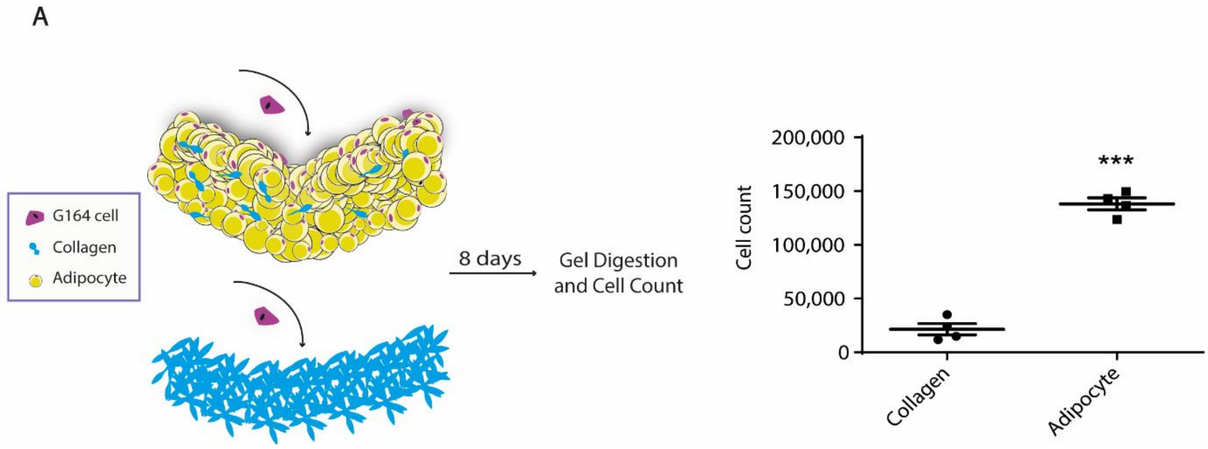

Supplemental Figure 5. Adipocytes increase G164 proliferation. Related to Figure 1. (A) G164 cells were cultured either on adipocyte gels or collagen gels for 8 days. After 8 days, gels were digested and the total cells present counted using an automated cell counter.
